# Supplementary figures and images for: Strain Typing of Classical Scrapie and Bovine Spongiform Encephalopathy (BSE) by Using Ovine PrP (ARQ/ARQ) Overexpressing Transgenic Mice
Source: Int J Mol Sci. 2022 Jun 16;23(12):6744. doi: 10.3390/ijms23126744 (PMC9223460; doi:10.3390/ijms23126744)

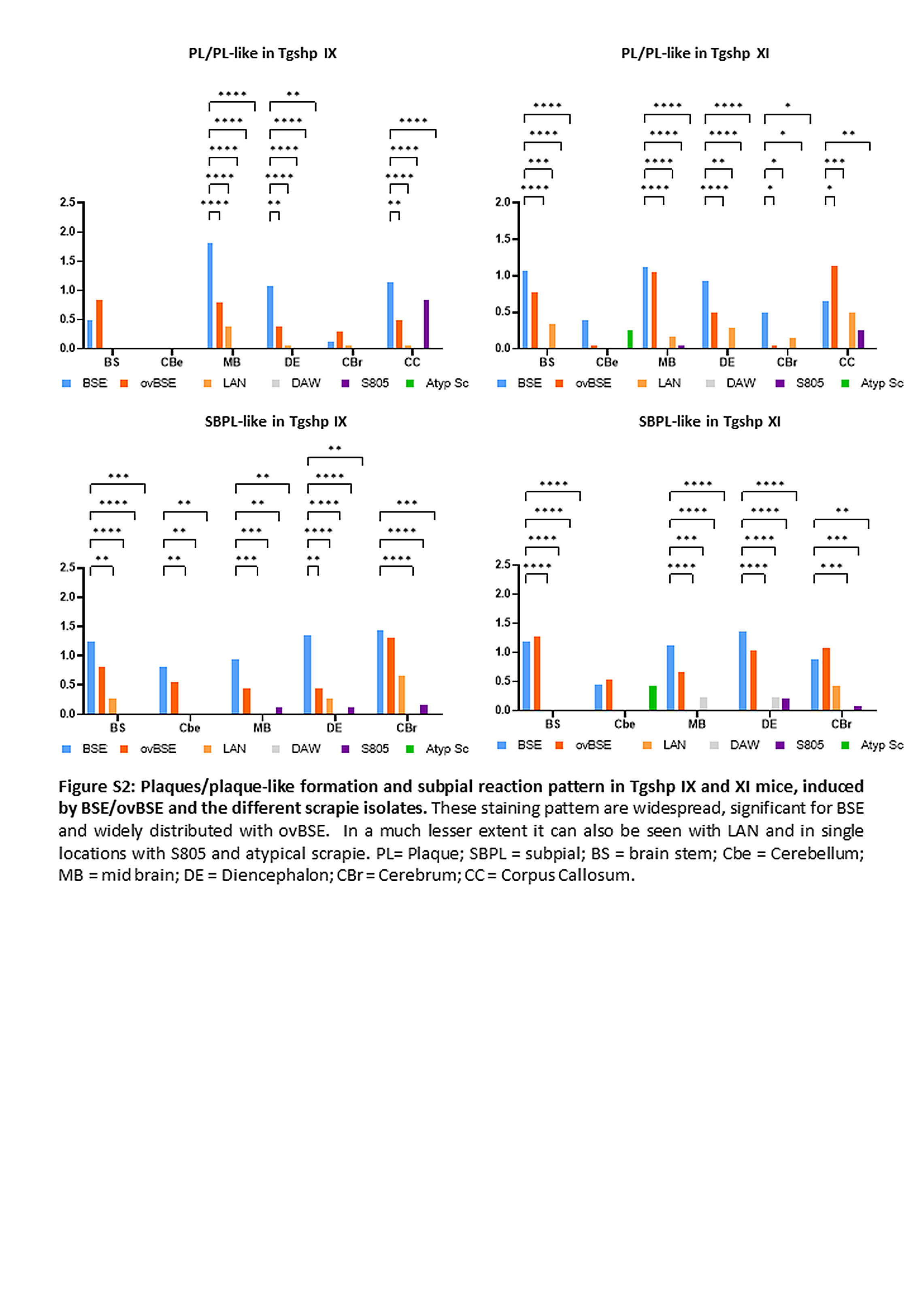

Supplement: Supplementary file 1 [file ijms-23-06744-s001.zip › Fatola et al Supl Fig S2-1.tif]

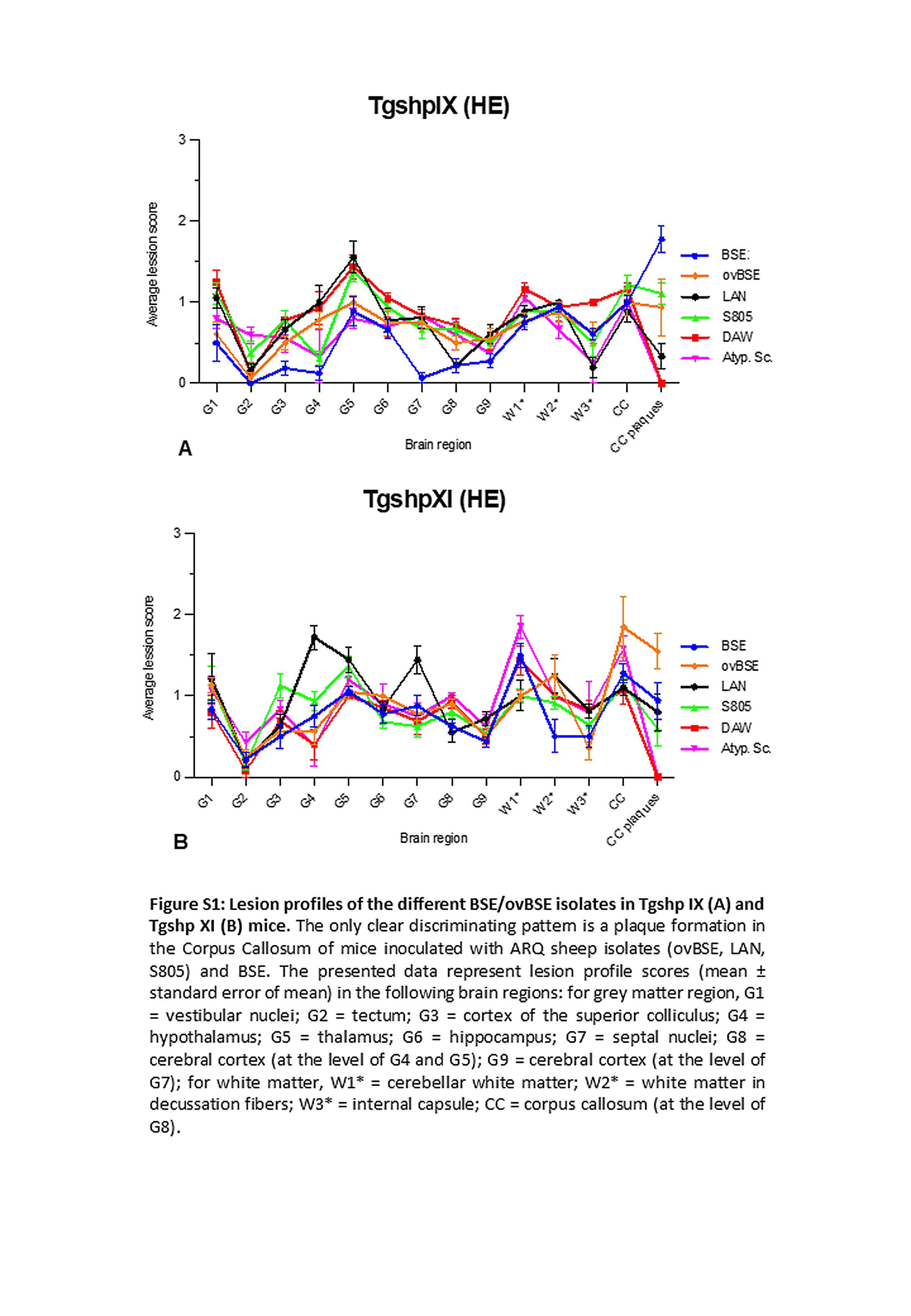

Supplement: Supplementary file 1 [file ijms-23-06744-s001.zip › Fatola et al Supll Fig S1-1.tif]
